# Weekend-Based Parent-Group Intervention to Reduce Stress in Parents of Children and Adolescents with Type 1 Diabetes: A Pilot Study

**DOI:** 10.1155/2019/7935945

**Published:** 2019-11-29

**Authors:** Lucia Ferrito, Barbara Predieri, Dorina Pjetraj, Maria Cristina Alessandrelli, Manuela Pagnini, Antonio Iannilli, Monica Marino, Stefano Tombolini, Basilio Pintaudi, Giuseppe Lucisano, Fabiana Zani, Lorenzo Iughetti, Antonio Nicolucci, Valentino Cherubini

**Affiliations:** ^1^SOD Pediatric Diabetology, Department of Women's and Children's, “G. Salesi” Children's Hospital, AOU Ospedali Riuniti Ancona, Italy; ^2^Department of Medical and Surgical Sciences of the Mother, Children, and Adults, University of Modena and Reggio Emilia, Modena, Italy; ^3^Pediatric Psychology, “G. Salesi” Children's Hospital, AOU Ospedali Riuniti Ancona, Italy; ^4^SSD Diabetes Unit, Niguarda Ca' Granda Hospital, Milan, Italy; ^5^CORESEARCH SRL-Center for Outcomes Research and Clinical Epidemiology, Pescara, Italy

## Abstract

Diagnosis of type 1 diabetes (T1D) in a child is often associated with anger, denial, fear, and depression from the parents. The aim of the study was to improve parents' adaptation to the diagnosis of diabetes of their child. Sixty-two parents (29 mothers, 33 fathers) of 36 children with type 1 diabetes (mean age = 11.3‐3.3 years; diabetes duration > 1 year; HbA1c = 57 ± 11 mmol/mol) participated in a three-day educational working group pilot intervention study. Intervention was based on the reexamination of the traumatic event of diagnosis of T1D through spatial and time-line anchorage, retracing of the future, emotional awareness, and interactive discussion. Relaxing technique, diaphragmatic breathing, and guided visualization were used by 2 psychologists and 1 pediatric endocrinologist. The study was approved by EC and participants filled a consent form. At baseline and after intervention, parents filled in a questionnaire including Diabetes-Related Distress (DRD), Parent Health Locus of Control Scale (PHLOC), Parent Stress Index Short Form (PSI-SF), Hypoglycemia Fear Survey-Parents (HFS-P) and Hypoglycemia Fear Survey-Parents of Young Children (HFS-P-YC), and Health Survey Short Form-36 (SF-36). Three months after the intervention, both parents reported a reduction in the “difficult child” subscale of the PSI-SF (*p* < 0.05) and increased scores of social functioning of the SF-36 (*p* < 0.05). DRD score was significantly reduced in mothers (*p* = 0.03), while the “parental distress” subscale of the PSI-SF was significantly improved in fathers (*p* = 0.03). This weekend-based parent group intervention seems to reduce stress and improve social functioning of parents of children and adolescents with type 1 diabetes.

## 1. Introduction

Type 1 diabetes (T1D) is a chronic disease that involves a rigorous daily self-care regimen including insulin administration, blood glucose monitoring, dietary management, and exercise [1]. Parenting challenges imposed by diabetes can be burdensome and, for some, overwhelming [2, 3]. In childhood, parents are required to assume the burden of daily diabetes self-care, while in adolescents, therapeutic and behavioral synergy between the parents and the child affected by the disease is required [4]. School-age children are also prone to wide-ranging fluctuations in blood glucose levels because of their irregularity in food intake and exercise and greater sensitivity to insulin, thus leading to an increased risk of hypoglycemia and ketoacidosis [5–7] . All of these factors combined may impact parents' functioning and their level of stress, which in turn may affect their ability to care for their child [8, 9]. In a systematic review, Whittemore et al. showed that the prevalence of parental psychological distress across all studies ranged from 10% to 74%, with an average of 33.5% of parents reporting distress at diagnosis of diabetes, while 19% of parents reporting distress 1 to 4 years after diagnosis [10]. In fact, even after a period of adaptation, where new routine is introduced and parents become more confident in diabetes management, parents are found to be in continuous surveillance, always in a state of bodily and mental readiness in order to prevent emergencies [11]. When compared with matched controls, parents of children with T1D showed higher parenting stress [12] and this negatively affects family communication [13], parenting competence, [14], and child psychological adjustment [15] and contribute to increase the risk of anxiety disorders and depressive disorders in caregivers [10].

Educational approaches have been developed to target learning and reinforce concepts related to the management of the disease. Educational camps for children and adolescents with T1D have proved useful in improving metabolic control, knowledge of the management of acute complications, quality of life (QoL), and other psychosocial outcomes [16]. Areas covered in these camps mainly concern clinical and management situations in which the child/adolescent is the focus. However, these educational camps do not target the point of view of parents, their feelings regarding their child's illness, and the impact this can have on their QoL. There is a wide variety of group therapy options available for type 1 diabetes patients; however, few interventions target specifically parents. In a pilot randomized controlled trial among parents of children and adolescents with diabetes type I using relaxation techniques, a statistically significant decrease in perceived stress, as well as in parenting stress, was registered in an intervention group compared to the control [17].

A family-centered, camp-based intervention in young children with T1D improved perceived QoL and stress in their mothers by the use of didactic and interactive parent education [18]. In a randomized trial, a structured behavioral group training program (DELFIN) was tested to reduce parenting stress and improve parenting skills [19]. Parents' psychological distress was reduced in both the intervention and control group, with no statistically significant difference between the groups. In this study, a new approach is proposed to reduce stress in parents by using an integration of relaxation, breathing, and visualization techniques, methods derived from neurolinguistic programming such as spatial anchoring, past-experience revival, and a short intervention of problem solving and experience sharing. Since we used the above experiential approach and spatial-temporal anchoring, we called this technique Body Emotional Maps (BEM). Parents underwent this path in groups, as group therapy is proven to provide the opportunity to empathize, overcome social isolation, promote hope, inspire perspectives, codiscover skills, and reinforce changes in others while gaining confidence to do the same [20]. Weekend camps have several advantages for participants: they are usually settled in a pleasant environment out of the hospitals, thus favoring relaxation and social relationship, and allow intensive learning. This pilot study was designed to assess the impact of a weekend-based parent group intervention with the BEM path on parents of children and adolescents with T1D. We explored the hypothesis that weekend camps could reduce parental stress and improve parents' psychosocial functioning.

## 2. Methods

### 2.1. Participants

Subjects were selected from a database of 500 children/adolescents with T1D belonging to the Unit of Diabetology of Ancona and Modena Pediatric Hospitals. Parents were considered eligible for the study if

1. their child was aged under 18 years at the time of recruitment and had been diagnosed with T1D for at least three years
2. parents have never been diagnosed as psychiatric patients based on self-reports in the course of a face-to-face interview
3. children have never been diagnosed with psychiatric or behavioral disorders
4. parents have never participated in any group intervention or weekend family meeting
5. parents were evaluated at risk of parental stress during the most recent clinical interview in the opinion of both the physician and the psychologist

One hundred and two parents were considered eligible based on the inclusion/exclusion criteria. Written communication was sent to the parents to present the program and encourage participation.

A total of thirty-five families were enrolled; twenty-seven were composed of two parents, and eight were single-parent families (six fathers and two mothers). In a family, there were 2 children with diabetes. We are presenting results from 33 fathers and 29 mothers. Subjects were randomly assigned to three camps settled on different weekends. The characteristics of the children and their parents are reported in Table 1. The total cost of the camp intervention was 24,000 €, including lodging, meals, staff time, and transportation covered by the volunteer organization.

### 2.2. Study Design and Procedures

The study protocol was reviewed by the local Ethics Committee. Written informed consent was obtained from study participants at baseline (i.e., before camp). Staff consisted of two psychologists, two physicians, and a counselor. Data was collected at baseline and one month (M1) and three months (M3) after the camp. At baseline, the following information was collected either from medical records and interview: gender, age, level of school education, body weight, height, duration of T1D, insulin treatment, presence of complications of diabetes, last value of glycosylated hemoglobin (HbA1c), frequency of self-monitoring of capillary blood glucose, presence of other chronic diseases, hospitalizations in the last year due to severe hypoglycemia, diabetic ketoacidosis or other reasons, previous participation in school camps, and level of physical activity. Data collected from the parents included age, level of school education, employment status, marital status, number of children, number of people which the household comprised, and whether any other family members had a diagnosis of diabetes. HbA1c was measured locally at each participating center using the same method, DCA 200 Vantage, which is DCCT standardized.

### 2.3. Parent Group Intervention

Each weekend camp was held in a reserved, cozy, and intimate location, from Friday afternoon to Sunday morning. Working groups were planned to be composed by 20 parents; however, in one session a total of 22 parents were accepted, including two unpaired parents. The parent group intervention consisted of three group sessions alternated with leisure activities. The first session consisted of an overview of the program, introduction of participants, and division of subjects into two groups (max. 12 subjects) for subsequent sessions. This first session lasted two hours and was carried out by all members of the multidisciplinary team. The second session gave the parents the opportunity to share a free narrative of their emotional experience since their child's diagnosis to date. A psychologist and a counselor conducted each group. Specific techniques derived from neurolinguistic programming were combined under the name of Body Emotional Maps (BEM) and proposed in this session. These included the following techniques: future pacing, a type of mental imagery to anchor or connect changes and resources to future situations or a particular event; spatial anchoring, which involves creating a link between a space and a state using a physical space as a trigger for states; and sensorial identification, which consists of identifying somatic sensations correlated to the emotional state. This session lasted four hours and was alternated with breaks. In the third session, which lasted 2 hours, each participant was encouraged to identify obstacles and resources to reach the best possible adaptation to their child's illness and to share his/her experiences with the group. At the end of the weekend, a plenary session was carried out with staff and participants to acknowledge the main topics which emerged. Participants also shared with the group and the team feedback about the program and possible suggestions to improve future initiatives.

### 2.4. Study Questionnaires

Parents were asked to complete questionnaires available through an online platform designed for study proposals. Questionnaires had to be completed within a week before weekend camp took place (baseline), then one month (M1) and three months (M3) after the camp. The following questionnaires were administered to the parents: Parenting Stress Index Short Form (PSI-SF), Diabetes-Related Distress (DRD) questionnaire, Parent Health Locus of Control Scale (PHLOC), Health Survey Short Form-36 (SF-36), and, depending on the age of the child, Hypoglycemia Fear Survey-Parents of Young Children (HFS-P-YC) or Hypoglycemia Fear Survey-Parents (HFS-P).

#### 2.4.1. Parenting Stress Index Short Form (PSI-SF)

The Parenting Stress Index assesses parental stress in the areas of parental distress, stresses from interaction with the child, and stresses from a child's behavioral characteristics [21–24]. The short form consists of 36 items divided into three subscales: (1) the Parental Distress (PD) subscale (12 items) assesses a parent's distress that results as a function of parenting; (2) the Parent-Child Dysfunctional Interaction (P-CDI) subscale (12 items) assesses the parent's perception that the child does not meet the parent's expectations and the perception that the interactions with the child are not reinforcing to the parent; and (3) the Difficult Child (DC) subscale (12 items) assesses the extent to which children have behavioral characteristics that make them easy or difficult to manage. The subscale scores range from 12 to 60, and the Total Stress subscale scores ranges from 36 to 180, with higher scores indicating greater levels of parental stress. Thus, responses higher than the 85th percentile are interpreted as “clinically significant” for high levels of family stress. In addition, the measure provides a Total Stress score, which is the sum of PD, P-CDI, and DC. Finally, it is possible to calculate the Defensive Responding scale, which derives from seven items contained in the Parental Distress subscale. It assesses the extent to which the subject responds with a strong bias to present a favorable impression of themselves in order to decrease the appearance of problems or stress in the parent-child relationship. In the presence of extremely low scores, with a raw score of 10 or less, results should be interpreted with caution.

#### 2.4.2. Diabetes-Related Distress (DRD)

The Diabetes-Related Distress questionnaire, already used in previous studies [25, 26], was specifically adapted to assess the level of distress felt by the parents in relation to the T1D of their child. The emotional impact of T1D (diabetes-related distress) measures the specific concern of the patient's parent and the negative emotions related to the child's illness. The questionnaire consisted of five questions. Parents were requested to report how many times in the previous 4 weeks they experienced certain emotions using a Likert scale from 1 (always) to 5 (never). The total score is calculated by the sum of the values of each item, after reverting the individual values. The final score ranges from 0 to 100, with higher values indicating higher levels of distress.

#### 2.4.3. Parent Health Locus of Control Scale (PHLOC)

The PHLOC is a questionnaire consisting of 28 items directed at assessing the parents' opinions about the health of their child [27]. The questionnaire aims to identify the aspect that has the greatest impact on the child's health according to their parents. These aspects include concern about the child themselves (5 questions), divine influence (3 questions), fate/luck (5 questions), mass media (3 questions), parents (7 questions), and professional (5 questions). Parents express their degree of agreement or disagreement with each statement using a Likert scale from 1 (strongly disagree) to 6 (completely agree). For each dimension a score is calculated, derived from the sum of the values for each item belonging to the subscale. The Italian version of the questionnaire was validated in 2009 [28].

#### 2.4.4. Hypoglycemia Fear Survey-Parents (HFS-P) and Hypoglycemia Fear Survey-Parents of Young Children (HFS-P-YC)

The HFS-P is a questionnaire consisting of 26 items and is aimed at parents of children/adolescents aged between 6 and 18 years [29]. This measure takes less than 10 minutes to complete and can be scored quickly to provide a measure of fear of hypoglycemia. The HFS-P has two subscales that measure parents' behaviors related to preventing an episode of hypoglycemia and their worry that their child may experience a hypoglycemic episode. For each item, parents are asked to report how often the item is true for them using a 5-point Likert scale (“1=never” to “5=very often”). The HFS-P yields a subscale score for each of the Behavior and Worry scales and a Total Score, which was scored for each participant (possible range 26-130). Higher scores indicate greater fear of hypoglycemia. In the questionnaire HFS-P-YC, seven questions of the HFS-P have been reworded to be more appropriate for parents of children under 6 years of age [9]. The Italian version of the two questionnaires has been specifically translated for this study, which will also allow the validation of the instruments.

#### 2.4.5. Health Survey Short Form-36 (SF-36)

The SF-36 scale was widely used in many countries and many different clinical conditions for evaluating Health-Related Quality of Life [30–34]. It includes eight domains: physical function (10 questions), role limitations due to physical problems (4 questions), bodily pain (2 questions), perception of general health (5 questions), energy/vitality (4 questions), social functioning (2 questions), role limitations due to emotional problems (3 questions), and mental health (4 questions). The scores for each dimension of the questions are coded, calculated, and translated into a scale ranging from 0 (worst possible health status) to 100 (best possible state of health). The eight domains may be further grouped into two summary measures of the physical component summary (PCS) and the mental component summary (MCS). These aggregated scores are converted into norm-based scores (mean: 50; SD: 10), and higher scores indicate more favorable physical functioning and psychological well-being.

#### 2.4.6. Study Endpoints

The primary end point of the study is represented by the longitudinal change between the values recorded at baseline and at M3 in the total stress score (PSI-SF) of the parents.

Secondary end points are the longitudinal changes between the values recorded at baseline, M1, and M3 of the scores of the following scales and respective subscales: DRD, PHLOC, HFS or HFS-P-YC, and SF-36. Moreover, HbA1c levels of children were measured at each time point.

### 2.5. Statistical Considerations

The change in QoL can be expressed in terms of effect size (value at the end of the study − the value at baseline/standard deviation of the measure at baseline). An effect size of 0.5 or greater can be regarded as clinically relevant [26]. In order to detect an effect size of at least 0.5 with a statistical power of 80% (*α* = 0.05), 66 parents had to be enrolled. The descriptive characteristics are summarized as mean and standard deviation (SD) in the case of continuous variables and percentages for categorical variables. The comparisons between the scores of the questionnaires at baseline, M1, and M3 are based on mixed models for repeated measures. Questionnaire scores are reported as estimated means with their standard error (SE). Analyses were performed on the whole sample as well as separately for mothers and fathers. The correlation between changes in the PSI-SF score and changes in the other scores was assessed through the Spearman correlation coefficient. Independent correlates of changes in the PSI-SF score were assessed through multiple regression analysis with changes in the PSI-SF score (score at M3—score at baseline) as the dependent variable. The following covariates were tested in the model: children's age, duration of T1D, last HbA1c value, insulin therapy (CSII vs. MDI), parent's age, gender, level of school education, employment status, and number of people in the household. Results are expressed as beta parameters and their relative *p* values.

## 3. Results

All subjects completed the required questionnaire at baseline, M1, and M3. Values of questionnaire scores at baseline, M1, and M3 are reported in Table 2.

### 3.1. Primary Endpoint

Results documented a significant improvement of the PSI-SF total score at M3 compared to baseline, from 75.4 ± 2 to 70.2 ± 2.5, *p* value = 0.03. This result was explained by a reduction in the “distress” subscale, from 25.8 ± 1.0 to 23.6 ± 1.0, *p* value = 0.03, and an even more pronounced reduction in the “difficult child” subscale, from 27.9 ± 0.9 to 25.6 ± 0.9, *p* value = 0.004. Effect sizes for both subscales indicate a moderate intervention effect.

### 3.2. Secondary Endpoint

A significant improvement in the “social functioning” SF-36 domain, both at M1 and M3, was also documented (from 75.6 ± 2.4 at baseline to 80.4 ± 2.5 at M1, *p* value = 0.03, and 83.1 ± 2.4 at M3, *p* value = 0.007). Effect size for this subscale indicates a moderate intervention effect. Finally, the scores relative to the “divine influence” and “fate/luck” subscales of the PHLOC significantly increased at M1, but the changes were no more significant at M3. No significant changes were documented for the other scales or subscales utilized. HbA1c levels of children did not substantially change from baseline to M3 (7.37 ± 1.1 vs. 7.34 ± 1.1%, respectively, *p* = 0.76). At baseline, a significant correlation was found between higher levels of parenting stress and diabetes-related distress, fear of hypoglycemia, and poorer psychological well-being. Parents' level of school education and type of insulin delivery did not correlate with changes in stress scores. No other significant difference between baseline and M3 was reported.

Higher levels of diabetes-related distress were also significantly associated with fear of hypoglycemia and poorer psychological well-being (Table 3). A significant correlation was found between the reduction in the PSI-SF total score and an increase in the SF-36 Mental Component Summary score (Spearman's *R* = 0.41; *p* = 0.0008). No additional significant correlation between changes in PSI-SF and changes in the other scores was detected.

### 3.3. Differences between Mothers and Fathers

**Figure 1:**
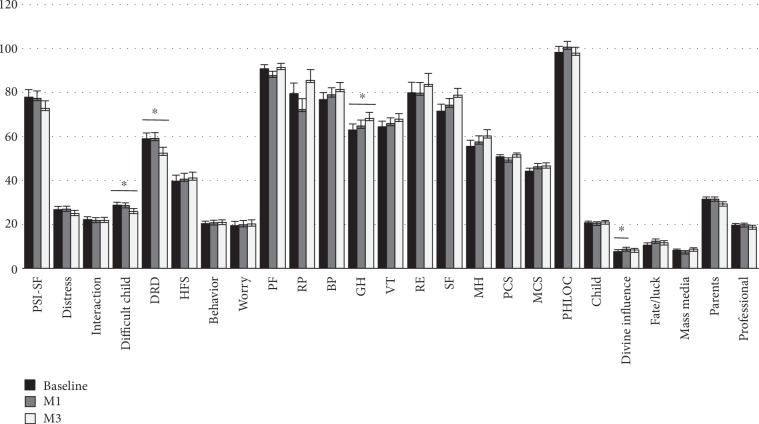
Estimated mean scores in mothers. Asterisks indicate significant differences between species (planned comparisons; ^∗∗∗^*p* < 0.05). Abbreviations: M1—after one month; M3—after three months; PSI-SF—Parenting Stress Index Short Form; DRD—Diabetes-Related Distress; HSF—Hypoglycemia Fear Survey; PF—physical function; RP—role limitations due to physical problems; BP—bodily pain; GH—general health; VT—energy/vitality; RE—role limitations due to emotional problems; SF—social functioning; MH—mental health; PCS—physical component summary; MCS—mental component summary; PHLOC—Parent Health Locus of Control Scale.

**Figure 2:**
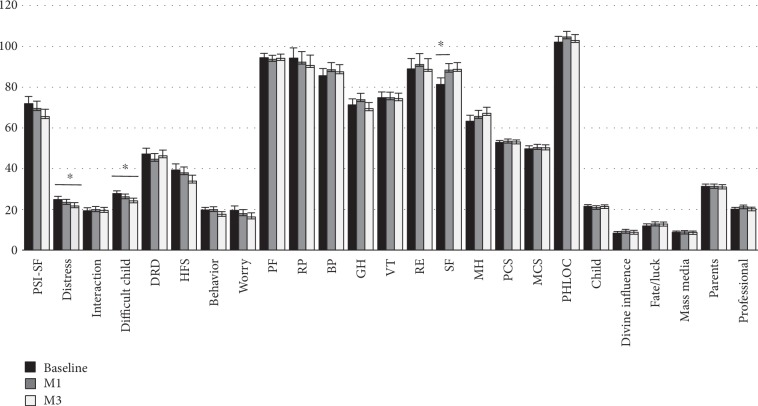
Estimated mean scores in fathers. Asterisks indicate significant differences between species (planned comparisons; ^∗∗∗^*p* < 0.05). Abbreviations: M1—after one month; M3—after three months; PSI-SF—Parenting Stress Index Short Form; DRD—Diabetes-Related Distress; HSF—Hypoglycemia Fear Survey; PF—physical function; RP—role limitations due to physical problems; BP—bodily pain; GH—general health; VT—energy/vitality; RE—role limitations due to emotional problems; SF—social functioning; MH—mental health; PCS—physical component summary; MCS—mental component summary; PHLOC—Parent Health Locus of Control Scale.

The analysis was also conducted separately for the two genders. Data relative to mothers (Figure 1) show a significant decrease in the PSI-SF “difficult child” subscale (from 28.8 ± 1.3 at baseline to 25.9 ± 1.3 at M3, *p* value = 0.04) and DRD score (from 59.0 ± 2.6 at baseline to 52.4 ± 2.7, *p* value = 0.03), and an increase in the SF-36 “general health perception” (from 62.9 ± 2.8 at baseline to 68.2 ± 2.8, *p* value = 0.05). A significant increase in the PHLOC “divine influence” was also documented at M1. As for fathers, a significant decrease in the “distress” (from 24.9 ± 1.5 to 21.8 ± 1.5, *p* value = 0.04) and “difficult child” subscales of the PSI-SF were documented at M3 (from 27.8 ± 1.3 to 24.2 ± 1.3*p* value = 0.02) and a significant improvement in the SF-36 “social functioning” domain was documented at M1 (from 81.3 ± 3.2 to 88.3 ± 3.2, *p* value = 0.03) (Figure 2). Finally, a multiple regression analysis was conducted to evaluate correlates of changes in the PSI-SF score (Table 4). The reduction in the PSI-SF score was inversely related to baseline children's HbA1c levels. Increasing parent's age was also associated with a marginally significant reduction in parenting stress. Parents' level of school education and type of insulin delivery did not correlate with changes in stress scores.

### 3.4. Parents' Satisfaction

At the end of the weekend, 60 subjects out of 62 expressed a high level of satisfaction for the intervention, and all but one stated that they would repeat the experience and suggest it to another parent with a similar situation. In addition, we gathered some other qualitative feedback from the parents after this experience. A father reported to us a very positive feedback in his family after the BEM path and now he ideally divides his life in two phases, before and after the BEM path; he said, “before BEM path I saw my son as a sick child, now I see my son as a healthy child.”

## 4. Discussion

The results of this study suggest that a weekend-based parent group intervention can be useful to reduce parental stress of children and adolescents with T1D. Three months after the camp, parents showed a significant decrease in parental stress, as measured by the PSI-SF. This result was obtained through a reduction in parental distress (PD) which includes an impaired sense of competence with parenting, stress resulting from restrictions on other life roles, conflict with the child's other parent, lack of social support, and depression. Moreover, significant improvement in the social functioning domain of SF-36 was observed one month and three months after the intervention, thus indicating that an improvement in social functioning may have played a role in reducing distress. The significant reduction in the difficult child subscale score further indicates that the level of parental stress was reduced through a decrease in the perception of difficulties in managing the child. Furthermore, a significant correlation was found between the reduction in parental stress and an increase in the Mental Component Summary Scale of SF-36, thus suggesting the role of a reduction of anxiety or depression influencing the level of stress.

One explanation for these results may derive from the process parents were guided through. They experienced different verbal and sensorial stimuli, which allowed them to focus on two crucial moments of their children's life: the initial trauma of the new diagnosis and the subsequent adaptation. Adaptation in particular has a major impact on chronic disease health outcomes [35]. Integration of different techniques and strategies such as relaxation techniques, guided imagery, and group sharing guided by a psychologist and a counselor helped parents find the internal resources to reach a higher sense of self-efficacy and lessen stress levels.

It is interesting to notice that both mothers and fathers registered a reduction in the “difficult child” scale, but only mothers had a significant improvement in “general health subscale”. As shown in previous studies, the burden of managing children with diabetes is often handled by mothers. Their caring role in preparing on meals, checking blood glucose levels, and administering insulin is crucial. All these duties expose them to a high level of stress [36, 37].

In literature, the relationship between parental stress and metabolic control remains unclear; two studies demonstrate no correlation [38, 39], while others suggest contrasting conclusions [40–42]. Although we could not assess the impact of reducing parenting stress on metabolic control of the children, which would require a longer period of observation, in our study, families whose children presented with a higher HbA1c registered a significant reduction in the PSI-SF score. This indicates that poor metabolic control is an important source of stress, and in these families there may be more room for improvement.

Of note, no changes were observed regarding fear of hypoglycemia, which can substantially contribute to parenting stress. In fact, in a study from Harrington et al., the most common concern of parents was preventing the low blood glucose of children, and this was supported by other studies [43, 44]. Our intervention did not specifically address the problem of hypoglycemia, and that gives reasons to our findings. In the future, it could be interesting to introduce structured courses about the use of technology in diabetes, carbocounting, and insulin dose adjustment alongside the intervention we proposed in the current study. This could increase the perception of self-efficacy which is associated with greater psychological well-being [45].

Finally, no major changes were detected as for the parental locus of control. Results suggest an increase in the reliance on external factors, such as divine interventions and fate, one month after the camp. Nevertheless, this effect was transient and disappeared after three months. In a pilot randomized trial, the impact of a stress management program (progressive muscle relaxation combined with diaphragmatic breathing) was assessed in reducing perceived stress and parenting stress, and increasing internal locus of control [17]. At the end of the intervention, lasting eight weeks, a significant reduction was documented in perceived stress and parenting distress in the intervention group as compared to baseline. A statistically significant difference between the two groups after the intervention was found only in perceived stress.

In comparison with other experiences described in literature, the group intervention tested in the present study relied on an intensive group program based on a single weekend, which was aimed at reducing parental stress mainly through the sharing of emotional experience and coping strategies. In our weekend program, the inclusion of techniques derived from neurolinguistic programming in a group setting could possibly have contributed to making the social experiment more engaging and accelerated the parent's ability to positively adapt to their child's illness.

This study has some limitations. It was a pilot study, therefore a control group was not included. It can thus be speculated that results depend on a generic “trial effect,” rather than the specific features of the weekend group intervention. On the other hand, we documented an improvement only on those aspects related to parenting stress and diabetes-related distress, which were the specific focus of the intervention. No major changes were documented in the other scales. Whether the positive results on stress are maintained over time is not known. In addition, parental fear of hypoglycemia was not investigated during this weekend program which is recognized to be a strong correlate with the distress. A revision of the approach, to also include these aspects could at least in theory further improve its impact on parents' QoL. Parents diagnosed with a psychiatric disorder were considered an exclusion criterion, as mental illness could to be a confounding factor for the aim of the study. Further studies may focus on this specific group of parents.

In conclusion, this pilot study supports the feasibility of the parenting program using the weekend-based parent group intervention. The first set of data suggests that it is possible to reduce parenting stress and diabetes-related distress in parents of young children and adolescents with T1D. It will be useful to confirm these findings with a randomized study. If these findings will be confirmed, it will be possible to plan further measures in an out-of-hospital context to help parents of children with diabetes manage stress.

## Figures and Tables

**Table 1:** Characteristics of children/adolescents and their parents.

Characteristics	No. (%) or mean ± SD
Age of children (years)	11.3 ± 3.3 (range 3-17.9)
Children's gender (males)	15 (41.7)
Duration of diabetes	5.7 ± 3.7
HbA1c (%)	7.4 ± 1.1
Treatment	
CSII	16 (44.4%)
MDI	20 (55.6%)
Mother age (years)	43.7 ± 5.7
Father age (years)	46.6 ± 6.2
Mother occupation	
Employed	28 (77.8%)
Unemployed	1 (2.8%)
Retired	7 (19.4%)
Mother school education	
Low school	11 (30.6)
Middle school	19 (52.7)
High school	6 (16.7)
University degree	—
Father occupation	
Employed	34 (94.4%)
Unemployed	1 (2.8%)
Retired	1 (2.8%)
Father school education	
Low school	1 (2.8)
Middle school	12 (33.3)
High school	20 (55.6)
University degree	3 (8.3)

**Table 2:** Estimated mean scores in the whole sample.

Scale	Baseline	M1	M3	P (M1 vs. baseline)	P (M3 vs. baseline)	Effect size M1 vs. baseline	Effect size M3 vs. baseline
PSI-SF	75.4 ± 2.5	74.5 ± 2.5	70.2 ± 2.5	0.64	0.03	0.4	0.4
PD	25.8 ± 1.0	25.3 ± 1.0	23.6 ± 1.0	0.54	0.03	0.5	0.5
I	21.0 ± 0.9	21.3 ± 0.9	21.0 ± 0.9	0.69	0.96	-0.3	-0.3
DC	28.5 ± 0.9	27.9 ± 0.9	25.6 ± 0.9	0.39	0.004	0.7	0.7
DRD	53.3 ± 2.0	53.0 ± 2.0	49.9 ± 2.0	0.88	0.13	0.1	0.1
HFS	40.1 ± 2.0	40.2 ± 2.0	38.7 ± 2.0	0.92	0.51	-0.1	-0.1
Behavior	20.0 ± 0.9	20.5 ± 0.8	19.5 ± 0.9	0.49	0.66	-0.6	-0.6
Worry	20.1 ± 1.4	19.7 ± 1.4	19.2 ± 1.4	0.75	0.54	0.3	0.3
SF-36							
PF	92.2 ± 1.5	90.2 ± 1.5	91.9 ± 1.5	0.09	0.82	1.3	1.3
RP	86.7 ± 3.6	81.1 ± 3.6	87.0 ± 3.6	0.14	0.96	1.6	1.6
BP	80.6 ± 2.4	82.4 ± 2.4	83.8 ± 2.4	0.44	0.27	-0.8	-0.8
GH	66.5 ± 2.0	68.4 ± 2.0	68.1 ± 2.0	0.21	0.42	-1.0	-1.0
VT	69.2 ± 1.9	70.0 ± 2.0	70.6 ± 2.0	0.62	0.50	-0.4	-0.4
RE	82.8 ± 3.7	84.3 ± 3.7	85.2 ± 3.7	0.63	0.53	-0.4	-0.4
SF	75.6 ± 2.4	80.4 ± 2.5	83.1 ± 2.4	0.03	0.007	-2.0	-2.0
MH	59.3 ± 2.1	60.9 ± 2.1	63.0 ± 2.1	0.35	0.09	-0.8	-0.8
PCS	51.8 ± 0.7	51.1 ± 0.7	52.1 ± 0.7	0.35	0.77	1.0	1.0
MCS	46.8 ± 1.0	48.1 ± 1.0	48.3 ± 1.0	0.14	0.18	-1.3	-1.3
PHLOC							
Child	100.9 ± 1.9	102.8 ± 1.9	100.8 ± 1.9	0.23	0.96	-1.0	-1.0
Divine	21.4 ± 0.5	20.8 ± 0.5	21.3 ± 0.5	0.29	0.92	1.2	1.2
Influence	7.9 ± 0.6	9.1 ± 0.6	8.5 ± 0.6	0.005	0.28	-2.0	-2.0
Fate/luck	11.1 ± 0.7	12.6 ± 0.7	12.1 ± 0.7	0.04	0.25	-2.1	-2.1
Mass media	8.8 ± 0.5	8.4 ± 0.5	9.1 ± 0.5	0.50	0.62	0.8	0.8
Parents	31.7 ± 0.7	31.2 ± 0.7	30.2 ± 0.7	0.48	0.08	0.7	0.7
Professional	19.9 ± 0.5	20.6 ± 0.5	19.6 ± 0.5	0.25	0.63	-1.4	-1.4

Abbreviations: M1—after one month; M3—after three months; PSI-SF—Parenting Stress Index Short Form; PD—Parental Distress; I—interaction; DC—difficult child; DRD—Diabetes-Related Distress; HSF—Hypoglycemia Fear Survey; SF-36—Health Survey Short Form-36; PF—physical function; RP—role limitations due to physical problems; BP—bodily pain; GH—general health; VT—energy/vitality; RE—role limitations due to emotional problems; SF—social functioning; MH—mental health; PCS—physical component summary; MCS—mental component summary; PHLOC—Parent Health Locus of Control Scale.

**Table 3:** Correlation between scale scores at time Baseline (Spearman's correlation coefficients and *p* values).

	DRD	HFS-P	PHLOC	SF-36 MCS	SF-36 PCS
PSI-SF	0.34 *0.008*	0.40 *0.001*	0.08 *0.55*	-0.49 *<0.0001*	0.09 *0.51*
DRD	—	0.44 *0.0003*	-0.02 *0.87*	-0.37 *0.003*	-0.21 *0.10*
HFS-P		—	0.12 *0.35*	-0.16 *0.20*	-0.22 *0.08*
PHLOC			—	0.08 *0.54*	-0.07 *0.58*
SF-36 MCS				—	0.008 *0.95*
SF-36 PCS					—

Abbreviations: PSI-SF—Parenting Stress Index Short Form; DRD—Diabetes-Related Distress; HSF—Hypoglycemia Fear Survey; SF-36—Health Survey Short Form-36; PCS—physical component summary; MCS—mental component summary; PHLOC—Parent Health Locus of Control Scale.

**Table 4:** Correlates of changes in PSI-SF score: results of multiple regression analysis.

Variable	Beta	*p*
Children's age (years)		
≤10	7.73	0.15
>10	r.c.	—
T1D duration (years)		
0-4	r.c.	—
4.1-7	4.53	0.51
>7	8.56	0.23
HbA1c (%)	-5.91	**0.02**
Insulin treatment		
MDI	r.c.	—
CSII	6.61	0.22
Parent's gender (M vs. F)	2.62	0.58
Parent's age (years)	-0.89	**0.05**
Level of school education		
Primary/secondary	r.c.	—
High school/university	0.97	0.85
Employment status		
Unemployed/retired	r.c.	—
Employed	-4.15	0.52
Number of family members	-0.07	0.98

## Data Availability

The data used to support the findings of this study are available from the corresponding author upon request.
